# Pain response in single-fraction 8-Gy radiotherapy for painful non-bone-metastasis tumors: a single-center retrospective study

**DOI:** 10.1093/jrr/rrae025

**Published:** 2024-05-07

**Authors:** Nobuki Imano, Takashi Kosugi, Kenta Konishi, Tetsuo Saito

**Affiliations:** Department of Radiation Oncology, Graduate School of Biomedical Health Sciences, Hiroshima University, 1-2-3, Kasumi, Minami-ku, Hiroshima-shi, Hiroshima 734-8551, Japan; Department of Radiation Oncology, Fujieda Municipal General Hospital, 4-1-11, Surugadai, Fujieda-shi, Shizuoka 426-8677, Japan; Department of Radiation Oncology, Fujieda Municipal General Hospital, 4-1-11, Surugadai, Fujieda-shi, Shizuoka 426-8677, Japan; Department of Radiation Oncology, Hamamatsu University School of Medicine, 1-20-1, Handayama, Chuo-ku, Hamamatsu-shi, Shizuoka 431-3192, Japan; Department of Radiation Oncology, Ariake Medical Center, 2600, Arao, Arao-shi, Kumamoto 864-0041, Japan

**Keywords:** radiotherapy, non-bone-metastasis tumors, single fraction, 8 Gy, pain response

## Abstract

The effectiveness of single-fraction 8-Gy radiotherapy for painful bone metastases has been verified in numerous randomized controlled trials. However, few reports have described the effectiveness of single-fraction 8-Gy radiotherapy in painful tumors other than bone metastases. We conducted a retrospective analysis to evaluate the pain response to single-fraction 8-Gy radiotherapy in painful non-bone-metastasis tumors. We included patients who had received single-fraction 8-Gy radiotherapy for such tumors between January 2017 and December 2022, excluding those with brain metastases, hematological tumors and those who received re-irradiation. Pain response assessment was based on the best responses documented in the medical records and conducted by two radiation oncologists. A total of 36 eligible patients were included in this study. The irradiation sites included primary lesions in eight patients, lymph node metastases in eight, muscle metastases in seven, pleural dissemination in four, skin/subcutaneous metastases in four and other sites in five. Pain response was assessed in 24 patients after radiotherapy. Pain response rate was 88% in evaluable patients; 21 of the 24 patients experienced response. The median assessment date for pain response was 37 days (range: 8–156 days) after radiotherapy. Re-irradiation was performed in four patients (11%). Single-fraction 8-Gy radiotherapy seemed to be a promising treatment option for painful non-bone-metastasis tumors and warrants further investigation.

## INTRODUCTION

Cancer patients with advanced, metastatic or terminal disease commonly experience pain, with a reported incidence of 66% [1]. Addressing cancer-related pain is crucial for enhancing the quality of life in these patients. Radiotherapy for bone metastases has long been established as an effective method for pain relief [2, 3]. A recent meta-analysis on radiotherapy for bone metastases indicated that a single 8-Gy irradiation is equally effective in alleviating pain compared with fractionated irradiation [2]. Accordingly, the European Society for Radiotherapy and Oncology Advisory Committee on Radiation Oncology Practice guidelines recommend that a single 8-Gy irradiation should be performed for painful bone metastases, considering its convenience and effectiveness [3]. Despite the widespread clinical use of radiotherapy for cancer pain caused by lesions other than bone metastases [4], high-quality reports are limited, and the optimal dose fractionation for non-bone-metastasis tumors is still unknown. Although a single 8-Gy irradiation, which has a strong evidence base for bone metastases, is suggested as a treatment option for non-bone-metastasis tumors at the expert-opinion level [5], there have been no reports on its efficacy. In the present study, we conducted a single-center retrospective analysis to evaluate the efficacy of a single 8-Gy irradiation for non-bone-metastasis tumors.

## MATERIALS AND METHODS

### Patient selection

We selected patients who received a single 8-Gy irradiation for pain palliation of painful tumors other than bone metastases at a single institution between January 2017 and December 2022. First, patients who met the following conditions were identified using the in-hospital system: (i) patients who received radiotherapy for symptom palliation; (ii) radiotherapy was performed from January 2017 to December 2022; (iii) irradiation to sites other than bone metastases and (iv) the painful tumor was not a brain metastasis. Next, we analysed medical records to identify patients who met the following conditions: (v) pain palliation was the main purpose of radiotherapy; (vi) the painful tumor was not a hematological tumor; (vii) no history of irradiation to the same site; (viii) radiotherapy to multiple sites for pain palliation was not planned during the same period; (ix) when the patient was irradiated more than once in the target period, the first irradiation was used for analysis and (x) patients received a single 8-Gy radiotherapy. Patients who simultaneously received radiotherapy to multiple sites were excluded to accurately assess the pain response.

This study was approved by the institutional ethical review board (approval number: R05-4).

### Treatment

External beam radiotherapy was delivered with 4–10 MV photons or electron beams using a 3D technique. The clinical target volume (CTV) was defined as the gross tumor volume. The planning target volume was defined by adding a 5–10-mm margin to the CTV. The patients received a single 8-Gy fraction.

### Data extraction

Pain response was evaluated based on the best response to radiotherapy assessed using the medical record description. The definition of pain response was based on the following medical record entries, which suggested the improvement of pain between before and after radiotherapy: (i) In the subjective records regarding the patient's statements, there was any description indicating that pain was either ‘improved’ or ‘reduced’ or had ‘disappeared’. (ii) In the objective records by the radiation oncologist, there was any description of either ‘pain reduction’, ‘pain disappearance’, ‘effective against pain’, or ‘good pain control’. (iii) In the numeric rating scale (NRS) records, the score decreased by 2 or more between before and after radiotherapy. Changes in the use of analgesics were not included in this definition of pain response. Two radiation oncologists, from which one was involved in the patient's treatment and the other not, independently conducted the evaluation based on the above definition; disagreements were resolved through discussion. Other data collected included age, sex, Eastern Cooperative Oncology Group performance status, primary site, irradiation site, survival time, date of response assessment and re-irradiation. To address the potential confounding factors influencing the pain response to radiotherapy, the following patient-related data were collected: use of opioids and adjuvant analgesics before treatment and on the day of pain response assessment, change in chemotherapy, surgical interventions for pain and nerve blocks.

### Statistical analysis

Overall survival was defined as the period from radiotherapy to death due to any cause. The Kaplan–Meier method was used to calculate survival. The median follow-up period was estimated using the reverse Kaplan–Meier method [6]. Toxicities were evaluated using the Common Terminology Criteria for Adverse Events version 5.0. Statistical analyses were performed using JMP Pro 16 software (SAS Institute Inc, Cary, NC, USA).

## RESULTS

### Patient and tumor characteristics

**Fig. 1:**
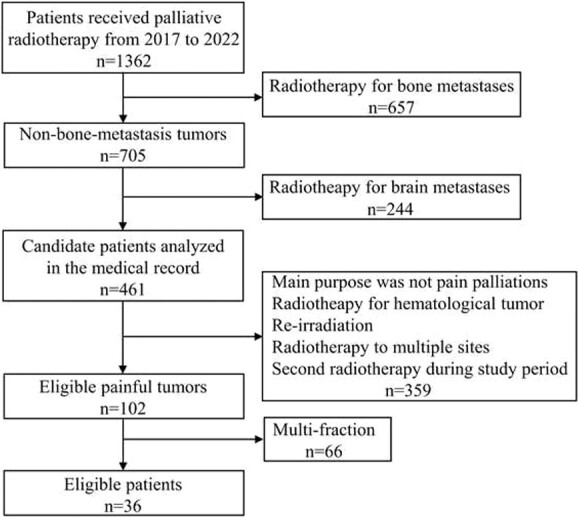
Enrollment characteristics of the study participants.

The enrollment characteristics of the study participants are shown in Fig. 1. Out of the 1362 patients who received palliative radiotherapy over the 6-year study period, 36 with painful tumors fulfilled the eligibility criteria. Patient and tumor characteristics of the 36 patients are shown in Table 1. The median age of the patients was 75 years, indicating an elderly population, and approximately three-fourths of the patients were men. Most tumors were lung cancers. Adenocarcinomas and squamous cell carcinomas each account for approximately one-third of the cases. All patients underwent 3D conformal radiotherapy.

**Table 1:** Patient and tumor characteristics

Characteristics	*n* = 36
Age (years) Median (range)	75 (26–91)
Sex, *n* (%) Male Female	27 (75) 9 (25)
ECOG-PS, *n* (%) 0 1 2 3	10 (28) 13 (36) 9 (25) 4 (11)
Primary tumors, *n* (%) Lung cancer Esophageal cancer Pancreatic cancer Others	26 (72) 2 (6) 2 (6) 6 (17)
Histology, *n* (%) Adenocarcinoma	12 (33)
Squamous cell carcinoma Small cell carcinoma Others Unknown	11 (31) 4 (11) 5 (14) 4 (11)
Irradiation site, *n* (%) Primary tumor Lymph node metastasis Muscle metastasis Pleural dissemination Skin/Subcutaneous metastasis Others	8 (22) 8 (22) 7 (19) 4 (11) 4 (11) 5 (14)

Abbreviations: ECOG-PS = Eastern Cooperative Oncology Group Performance Status.

### Pain response

The median follow-up was 13.6 months (95% confidence interval [CI] 3.6–23.5 months). Out of the 36 patients, pain response was evaluable in 24 (67%). Among them, 21 patients (88%) experienced a pain response to single-fraction 8-Gy radiotherapy. The median time to response was 37 days (range 8–56 days). Re-irradiation was performed in four patients (11%), and the intervals between initial irradiation and re-irradiation were 2.8, 3.5, 10.1 and 11.5 months, respectively.

### Confounding factors for pain response to radiotherapy

Out of the 21 patients who achieved a pain response, the baseline dose (daily dose without rescue use) of opioid analgesics, assessed based on the oral morphine equivalent dose, remained unchanged in nine patients, increased in eight patients and decreased in four patients between the date of radiotherapy and that of pain response assessment. Of the three patients who did not experience a pain response, the baseline dose of the opioid analgesic remained unchanged in one patient and increased in two patients. The total analgesic use could not be evaluated because rescue analgesic use was not assessable. Changes of chemotherapy between the date of radiotherapy and that of pain response assessment were confirmed in four patients who achieved a pain response and in one patient who did not. Adjuvant analgesics were initiated between radiotherapy and the date of pain response assessment in only two patients with pain response. No surgeries or nerve blocks were administered to any of the patients between radiotherapy and the date of pain response assessment.

### Toxicity

No acute toxicities of grade ≥ 3 were observed in the 24 evaluated patients.

### Survival rate

**Fig. 2:**
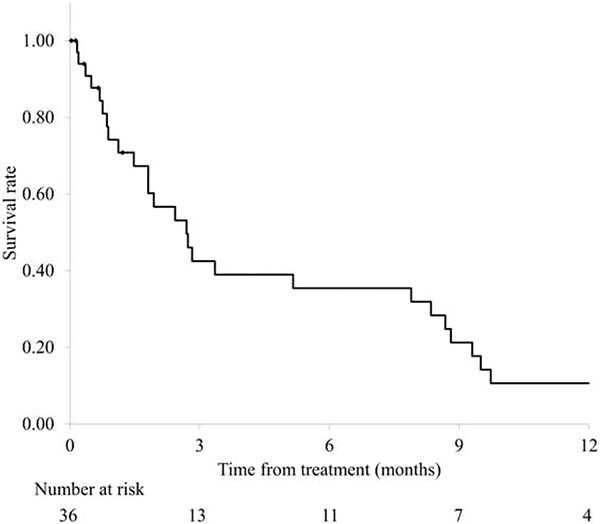
Overall survival for all patients.

In total, 27 deaths were recorded. The median survival time was 2.7 months (95% CI 1.6–3.8 months), and the survival rates at 6 months and 1 year were 35 and 11%, respectively (Fig. 2).

## DISCUSSION

We found that the response rate to single-fraction 8-Gy radiotherapy for painful non-bone-metastasis tumors was high at 92% in evaluable patients from a single-center retrospective analysis.

Alleviating cancer pain is important for improving the quality of life in patients with metastatic, advanced or terminal disease. Although there are many reports on the efficacy and optimal dose fractionation of external beam radiotherapy for bone metastases [2, 3], research on painful tumors other than bone metastases remains insufficient. Regarding limited reports on single-fraction radiotherapy for painful non-bone-metastasis tumors, a phase I/II trial conducted on 14 patients with recurrent ovarian cancer investigated the effectiveness of a single 7 Gy fraction or 6 Gy/2 fraction/1 day, and reported a high efficacy of complete response in five patients and partial response in seven (response rate: 86%) at a bioequivalent dose lower than a single 8 Gy dose [7]. In a retrospective report on re-irradiation of 78 patients with chest lesions related to lung cancer, 67 patients (86%) showed improvement in NRS 2 or better after single-fraction 8-Gy radiotherapy [8]. To the best of our knowledge, the present study is the first report on the initial irradiation of single-fraction 8-Gy radiotherapy for painful non-bone-metastasis tumors. Based on the aforementioned reports and our present study, a single 8-Gy irradiation can be considered a useful treatment option not only for bone metastases but also for non-bone-metastasis tumors.

The International Consensus Pain Response Endpoints (ICPRE), which considers both pain intensity and analgesic use, is recommended for evaluating the pain response in palliative radiotherapy [9]. A meta-analysis of radiotherapy for painful bone metastases using ICPRE reported lower pain responses than previous studies [10]. In the assessment of pain response based on the ICPRE, a response is not considered valid if the analgesic dose increases compared with the dose at the start of radiotherapy, even when the intensity of pain is reduced. Consequently, the response rate tends to be lower when evaluating response based on ICPRE than when evaluating response based on pain alone (as in the present study). In our study, we assessed the use of baseline opioid analgesic. However, because of the retrospective nature of the study, we were unable to evaluate the total analgesic dose, including rescue doses. Consequently, pain responses could not be determined using ICPRE. Given that the baseline opioid dose increased in 8 of the 21 patients who achieved a pain response, some of the patients who responded in our study would not have experienced a response if the response was assessed based on ICPRE.

Previous studies on radiotherapy for painful bone metastases have indicated a median survival of ~7–9 months from radiotherapy [11–15]. In the present study, a single 8-Gy irradiation was adopted in one-third of the eligible patients, which could have potentially involved patients with poor prognosis. Indeed, the median survival time was as short as 2.7 months. Although the present study does not indicate whether the prognosis of patients with painful non-bone-metastasis tumors is inferior to that of patients with painful bone metastases, it is evident that a certain proportion of the former have a poor prognosis. Single-fraction 8-Gy radiotherapy is recommended for bone metastases, even in long-term survivors [16]; however, it should be more actively considered for patients with a poor prognosis, considering a shorter treatment period.

Our study had several limitations. First, this was a retrospective study that included a small number of patients. Second, the evaluation of the pain response was mainly based on medical records and not precisely evaluated using NRS, and the timing of evaluations was not consistent. Third, the influence of analgesics on the pain response was not fully assessed because rescue analgesic use was not assessable. Therefore, the present study suggests the potential effectiveness of a single 8-Gy irradiation for painful non-bone-metastasis tumors, and the findings need to be verified in larger prospective studies. Fourth, the retrospective nature of the study hampered the assessment of the nature of pain such as a neuropathic pain. Fifth, the painful tumors analysed were heterogeneous, and various primary and metastatic tumors were evaluated together. The analysis of each painful tumor was hampered by the small sample size, and future studies with larger sample sizes need to be conducted.

## CONCLUSION

In the present study, we conducted a single-center, retrospective analysis to evaluate the efficacy of a single 8-Gy irradiation for non-bone-metastasis tumors. We observed a high response rate without severe toxicity. Our promising results warrant further research.

## PRESENTATION AT CONFERENCES

The 36th Annual Meeting of the Japanese Society for Radiation Oncology.
